# Timing of early water intake post-general anaesthesia: a systematic review and meta-analysis

**DOI:** 10.1186/s12871-024-02520-x

**Published:** 2024-04-09

**Authors:** Suwan Dai, Lingyan Chen, Min Wu, Liangyou Guo, Rong Wang

**Affiliations:** 1 Zhejiang Chinese Medical University, Hangzhou, China; 2 The First Hospital of Jiaxing, Jiaxing, China

**Keywords:** Early water intake, General anaesthesia, Resuscitation period, Enhanced recovery after surgery

## Abstract

**Background:**

Early water intake has gained widespread attention considering enhanced recovery after surgery (ERAS). In the present systematic evaluation and meta-analysis, we assessed the effects of early water intake on the incidence of vomiting and aspiration in adult patients who received general anaesthesia on regaining consciousness during the resuscitation period.

**Objective:**

To systematically analyse the results of randomised controlled trials on early postoperative water intake in patients who underwent different types of surgery under general anaesthesia, both at home and abroad, to further explore the safety and application of early water intake and provide an evidence-based foundation for clinical application.

**Design:**

Systematic review and meta-analysis.

**Methods:**

To perform the systematic evaluation and meta-analysis, we searched the Web of Science, CINAHL, Embase, PubMed, Cochrane Library, Sinomed, China National Knowledge Infrastructure (CNKI), Wanfang, and Vipshop databases to identify randomised controlled trial studies on early water intake in adult patients who received general anaesthesia.

**Results:**

Herein, we included 10 publications with a total sample size of 5131 patients. Based on statistical analysis, there was no statistically significant difference in the incidence of vomiting (odds ratio [OR] = 0.81; 95% confidence interval [CI] [0.58–1.12]; *p* = 0.20; I-squared [I^2^] = 0%) and aspiration (OR = 0.78; 95%CI [0.45–1.37]; *p* = 0.40; I^2^ = 0%) between the two groups of patients on regaining consciousness post-general anaesthesia.

**Conclusion:**

Based on the available evidence, early water intake after regaining consciousness post-anaesthesia did not increase the incidence of adverse complications when compared with traditional postoperative water abstinence. Early water intake could effectively improve patient thirst and facilitate the recovery of gastrointestinal function.

**Supplementary Information:**

The online version contains supplementary material available at 10.1186/s12871-024-02520-x.

## Introduction

Enhanced recovery after surgery (ERAS) was first introduced in 1997 by Kehlet [1], who mentioned that despite advances in anaesthesia, surgery, and perioperative care, several major surgical procedures were persistently impacted by adverse stress reactions, leading to poor outcomes such as nausea and vomiting, gastrointestinal paralysis, pain, and prolonged recovery time. However, accelerated multidisciplinary and multimodal interventions in rehabilitation surgery with a series of evidence-based optimisation measures can reduce patient stress, substantially reduce postoperative complication rates and lengths of stay, improve postoperative quality of life, and reduce overall healthcare costs [2]. With advances in society and economic growth, patient demand for comfort care is becoming more urgent. A comfortable medical experience can reduce postoperative stress, enhance patient cooperation, and facilitate postoperative recovery, aligning with the ERAS concept.

Fluid management is an important component of ERAS, and the implementation of early postoperative water intake is a critical initiative to promote fluid management and a favourable condition that facilitates gastrointestinal function recovery and reduces adverse stress reactions [3, 4]. According to traditional concepts, patients are required to abstain from routine food and fluid intake In the early postoperative stage, and considering patient safety during the postoperative period, to avoid complications such as aspiration and vomiting due to incomplete water intake on regaining consciousness [5]. The incidence of thirst in post-surgery patients reportedly exceeds 70%, and it the most urgent and intense sensations experienced during the perioperative period [6, 7]. Prolonged thirst can lead to an inability to concentrate, negative emotions such as anxiety and irritability [8], even increase the risk of delirium and various complications [9], and seriously impact the patient’s medical experience [10], which is in direct conflict with the concept of comfort care.

Previously, water intake immediately after surgery was considered undesirable and influenced by various factors, and adequate fluid post-surgery was maintained intravenously. However, greater or overloaded intravenous fluid volumes administration on the day of surgery was found to be independently associated with delayed recovery of postoperative symptoms and increased risk of postoperative complications, which are detrimental to early postoperative recovery [11, 12]. With the emphasis on the ERAS concept, there has been a gradual focus and change in the implementation of early postoperative fluid intake. According to a nationwide survey, healthcare professionals were assessed to determine their understanding and perspectives on early oral fluid intake, and it concluded that patients are suggested to begin oral intake soon after their surgical procedures [13]. Therefore, patients should be encouraged to drink water as early as possible after regaining complete consciousness and to limit postoperative intravenous fluid therapy post-surgery [3].

Currently, early postoperative water intake is implemented in adult patients who received general anaesthesia. However, the precise timing for this intervention remains inconsistent across studies, which could be attributed to discrepancies in the type of surgery and outcome indicators of the study population, differences in the study results, and a lack of comprehensive evaluation. Therefore, in the present study, we aimed to systematically analyse the results of randomised controlled trials on early postoperative water intake in patients who underwent different types of surgery under general anaesthesia, both at home and abroad, to further explore the safety and application of early water intake and provide an evidence-based foundation for clinical application.

## Methods

The present study was registered in the International Prospective Register of Systematic Reviews (PROSPERO) under the registration ID CRD42023395782.

### Search strategy

We searched the Web of Science, Cochrane Library, PubMed, Embase, CINAHL, Sinomed, China National Knowledge Infrastructure (CNKI), WanFang, and VIP databases from initiation to 31 December 2022 to identify relevant reports; we then reviewed the references of the included literature. The search strategy was as follows: (1) early oral hydration, early oral fluid, early oral intake, early drinking water, and drinking water; (2) anaesthesia, general or anaesthesia recovery period, and post-anaesthesia; and (3) adult and adult patients.

**Fig. 1:**
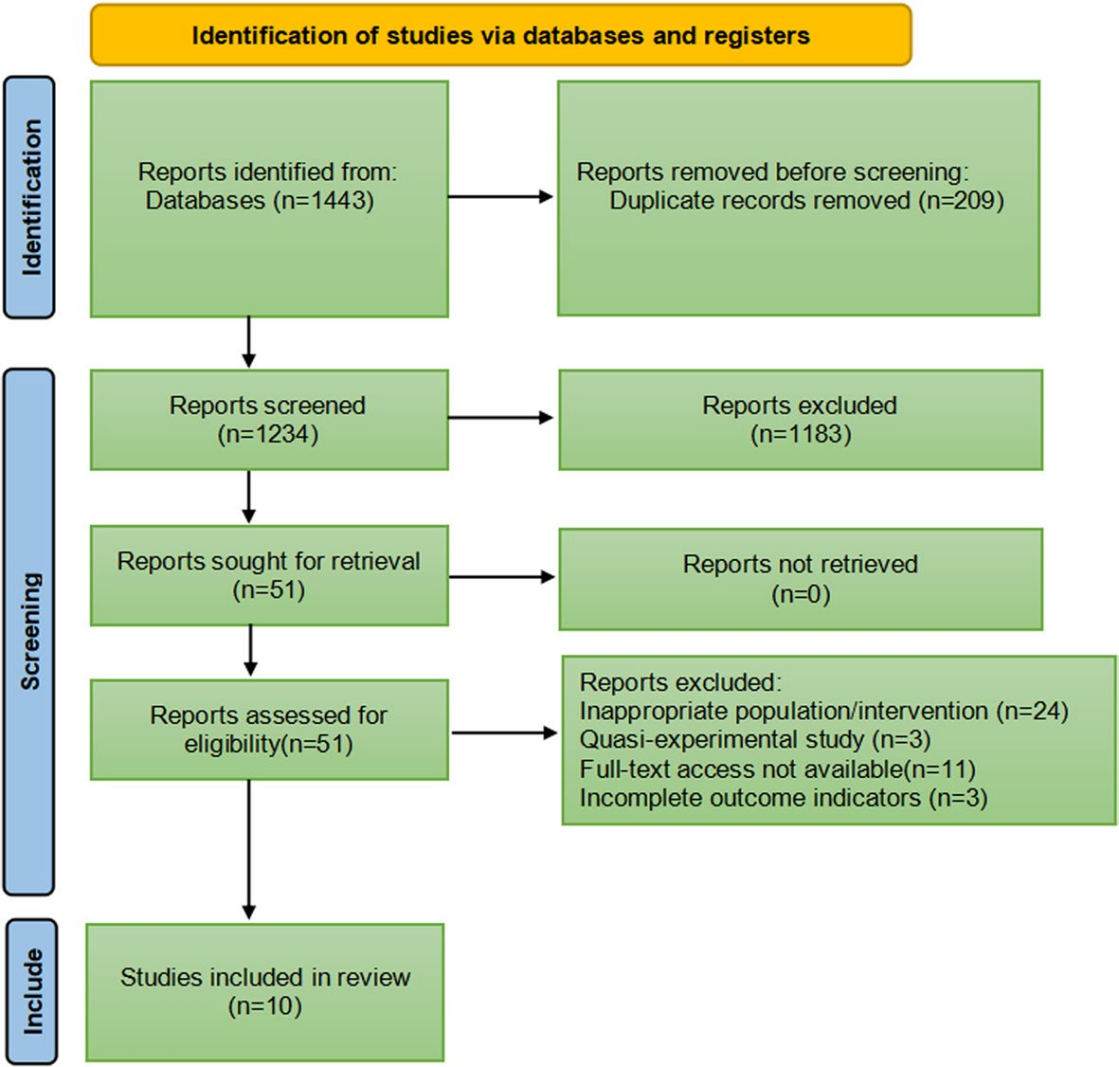
Search strategy flow diagram

Herein, two reviewers independently screened titles and abstracts of identified articles according to inclusion and exclusion criteria, subsequently reviewing the full text to determine eligible studies, as shown in Fig. 1. No language restrictions were imposed. In case of disagreements, the two reviewers conducted an initial discussion; if necessary, a third reviewer was consulted to reach a consensus. Information from the included studies was extracted and saved in Microsoft Excel. This study adheres to the Preferred Reporting Items for Systematic Reviews and Meta-Analyses 2020 statement, an updated guideline for reporting systematic reviews and meta-analyses.

### Inclusion and exclusion criteria

The inclusion criteria for eligible studies were as follows: (1) patients ≥ 18 years of age who underwent general anaesthesia; (2) patients who had received early oral hydration during recovery after general anaesthesia; (3) randomised controlled trials; (4) non-gastrointestinal surgery.

Exclusion criteria were as follows: (1) studies for which the full text could not be retrieved, or data could not be extracted completely; (2) case reports, reviews, dissertations, quasi-experimental studies, or multiple publications; and (3) studies that did not report relevant outcomes.

### Data extraction and study quality

Two reviewers independently extracted data and conducted quality evaluations to avoid bias. Discrepancies in interpretation were resolved by consensus or the involvement of a third reviewer. The following data were extracted using Microsoft Excel: author, year, sample size, type of surgery, intervention, and outcomes.

We assessed the risk of bias in randomised control trials using the Cochrane risk-of-bias tool based on six items: random sequence generation (selection bias); allocation concealment (selection bias); blinding of participants and personnel (performance bias); blinding of outcome assessment (detection bias); incomplete outcome data (attrition bias); selective reporting (reporting bias) and other bias. Each item was then judged based on three levels: “high risk”, “low risk”, and “unclear”. Moreover, the quality of the results was assessed using the GRADEpro software.

### Outcomes

After consultation, we determined that the primary outcomes were vomiting and aspiration rates. The secondary outcomes included nausea rate, thirst score, anal exhaust time, and first defaecation time.

### Study analysis

Meta-analysis of included studies was performed using Review Manager Version 5.3 software and Stata (version 16.0), and sensitivity analysis was performed according to the study quality to determine the stability of results. The mean difference (MD) and 95% confidence interval (CI) were used as effect sizes for continuous outcomes, and odds ratio (OR) and 95% CI were used as effect sizes for binary variables. The results of the meta-analysis are depicted as forest plots. The I-squared(I^2^) values was used to determine the heterogeneity between studies. An I^2^ value of < 50% was regarded as homogenous, and a fixed-effects model was selected. An I^2^ value of ≥ 50% indicated relatively moderate-to-high heterogeneity between studies, and we used the random-effects model for analysis. The causes of heterogeneity were further analysed, and subgroup analysis was conducted on factors that may lead to heterogeneity. Trial sequential analysis (TSA) was performed using Viewer software (version 0.9.5.10. beta) for the primary outcome to assess the risk of type 1 error caused by repeated testing.

## Results

**Fig. 2:**
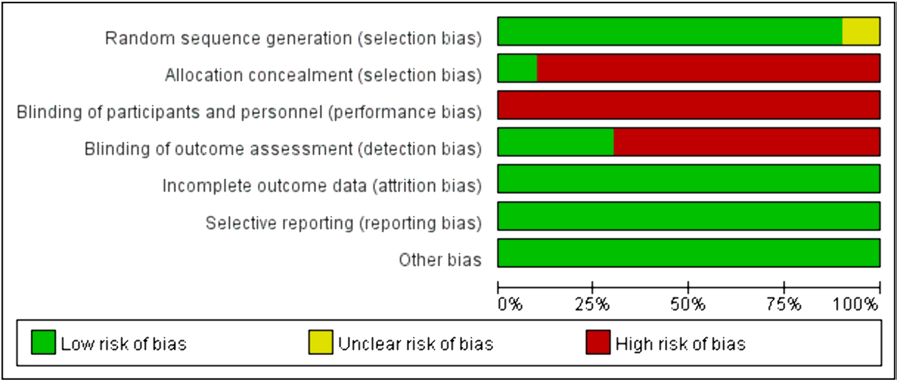
Risk of bias graph

**Fig. 3:**
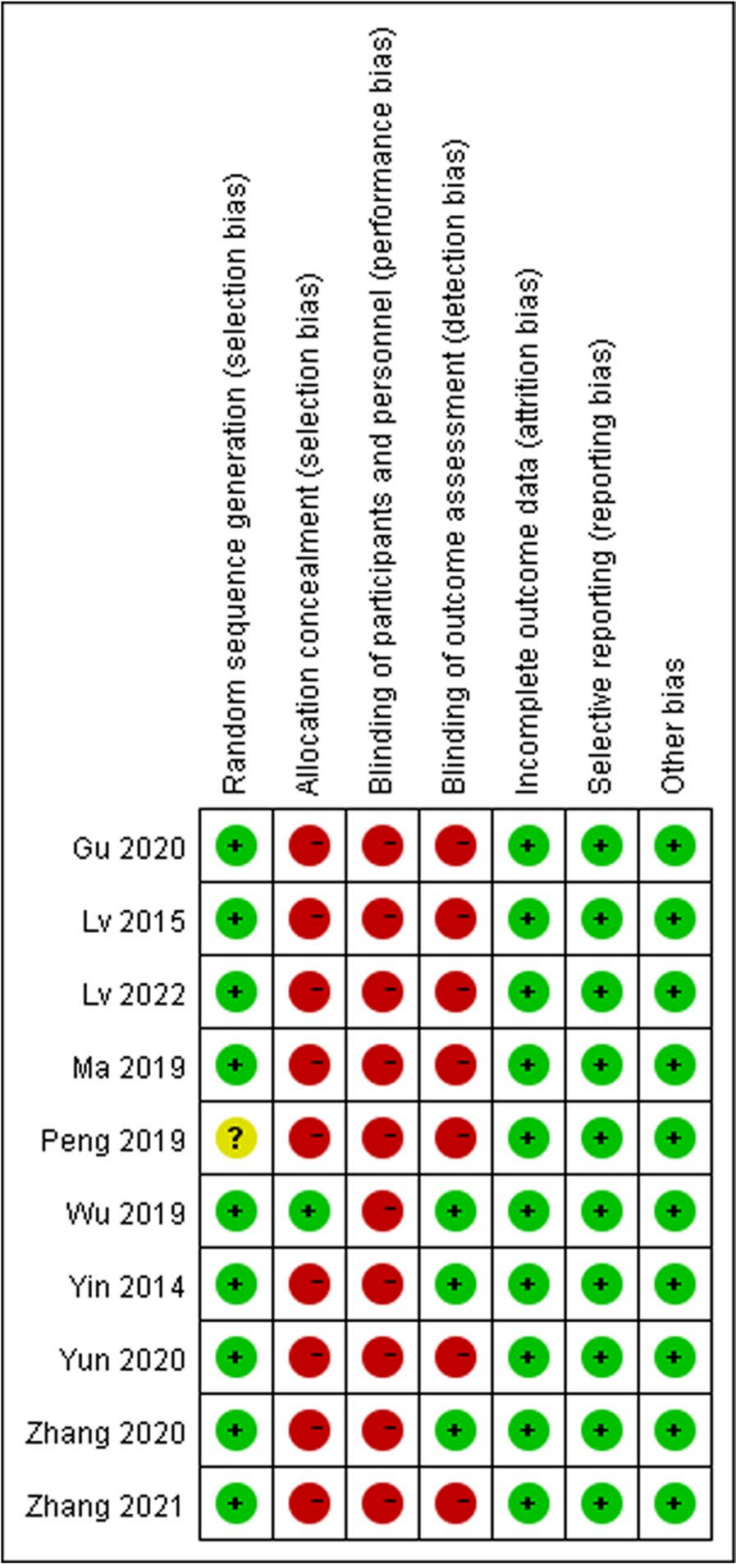
Risk of bias summary

In total, 1443 relevant studies were identified during the initial examination. After eliminating duplicate studies, 1234 studies were obtained. After screening the titles and abstracts, 51 studies were subjected to full-text screening, with 10 studies finally included [14–23], as shown in Fig. 1. The basic features of the included studies are presented in Table 1. Two studies were published in English [14, 15] and eight in Chinese [16–23], and all were randomised controlled trials with a total sample size of 5131 patients. Types of surgery included laparoscopic surgery, thoracoscopic surgery, and knee arthroscopy. Figures 2 and 3 present the bias risk assessment. The Cochrane Bias Risk tool reported a potentially ambiguous and high risk of bias, mainly in implementing blinding, given the challenges in double blinding for such procedural trials.

**Table 1:** Baseline characteristics of included studies

Authors	N	Inclusion criteria	Exclusion criteria	Type of operation	Intervention	Control	Vomiting	Aspiration	Nausea	Thirst	Anal exhaust time	Anal defecation time
Yin et al. (2014) [15]	1000 500, early drinking; 500, 4 h post-surgery	General anaesthesia	Delayed gastric emptying and existing gastrointestinal diseases; facial, oropharyngeal and throat surgery; impaired mental state; difficulty swallowing; gastrointestinal surgery; neurosurgery; thoracic surgery	Non-gastrointestinal general anaesthesia	After assessment, early water was given with a volume limit of 0.5 mL/kg.	Drinking water 4 h post-surgery	early drinking: 22/488 (5%) Regular: 20/495 (4%)	Not reported	early drinking: 37/488 (8%) Regular: 32/495 (6%)	early drinking: 46.27 ± 20.23 Regular: 61.09 ± 20.11	Not reported	Not reported
Wu et al. (2019) [14]	1735 867, early drinking; 868, 4 h post-surgery	General anaesthesia	Intestinal obstruction, dysphagia, diabetes, history of PONV, or American College of Anesthesiologists Class III or higher constitution	Laparoscopic cholecystectomy	After assessment, early water was given, and the total water volume was limited to 3 mL/kg.	Drinking water 4 h after surgery	early drinking:14/867(2%) Regular:31/868(4%)	Not reported	Early drinking: 23/867 (3%) Regular: 46/868(5%)	early drinking:37.51 ± 28.44 Regular:61.31 ± 33.21	Not reported	Not reported
Zhang et al. (2020) [19]	130 65, early drinking; 65, regular drinking water	General anaesthesia	Respiratory tract infection before surgery; a history of facial, oropharyngeal and laryngeal surgery; a history of dysphagia and gastrointestinal disorders; vertigo	Laparoscopic tubal plastic surgery	After assessment, early water intervention was administered, and the maximum water intake was 50 mL.	Regular drinking water	Not reported	Not reported	Not reported	Early drinking: 25/658 (38%) Regular: 38/58 (66%)	Early drinking: 12.00 ± 1.22 Regular: 15.10 ± 1.05	Not reported
Yun et al. (2020) [17]	268 134, early drinking; 134, regular drinking water	General anaesthesia	Mental illness or other complications; impaired speech and communication and use of sedatives and analgesics within 24 h; history of intestinal obstruction, dysphagia, diabetes, postoperative nausea and vomiting, inability to complete the assessment of various scales, or ASA level III or above	Thoracoscopic lobectomy	Early water intervention was performed after assessment, with no more than 3 mL for the first injection, 5–10 mL for each injection, and a total of no more than 2 mL/kg.	Regular drinking water	Early drinking:3/134 (2%) Regular: 2/134 (2%)	Not reported	Early drinking: 4/134 (3%) Regular: 5/134 (4%)	Early drinking: 29.4 ± 29.6 Regular: 63.3 ± 38.2	Not reported	Not reported
Peng et al. (2019) [20]	1000 500, early drinking; 500, regular drinking water	General anaesthesia	Gastrointestinal dysfunction; bowel and urine dysfunction; in-dwelling gastric tube; combined with digestive tract obstruction, digestive tract perforation, neurosurgery and oral surgery	Tracheal intubation under general anaesthesia	After assessment, 20 mL of warm sterilised water was given early, and the interval between two drinks exceeded 2 h.	Regular drinking water	Not reported	Early drinking: 19/500 (4%) Regular: 23/500 (5%)	Not reported	Not reported	Early drinking: 18.24 ± 7.79 Regular: 25.72 ± 10.33	Early drinking: 55.40 ± 13.96 Regular: 60.89 ± 13.55
Ma et al. (2019) [22]	80 40, early drinking; 40, regular drinking water	General anaesthesia	Gastrointestinal diseases; nausea and vomiting before the first intervention	Transnasal sphenoidal approach for pituitary tumour resection	Warm boiled water (10 mL) was given every 0.5 min within 1–2 h post-surgery, with no more than 0.5 mL/kg warm boiled water provided every 0.5 h within 3–6 h post-surgery.	Regular drinking water	Early drinking: 3/40 (8%) Regular: 5/40 (13%)	Not reported	Early drinking: 5/40(13%) Regular: 7/40 (18%)	Not reported	Early drinking: 16.53 ± 2.78 Regular: 19.70 ± 3.39	Not reported
Lv et al. (2015) [21]	200 100, early drinking; 100, regular drinking water	General anaesthesia	Oropharyngeal surgery; difficulty swallowing; airway obstruction	General anaesthesia	A small amount of water was given at the early stage, 5 mL 0.9% NaCl was given every 5 min, and the total amount should not exceed 0.5 mL/kg body weight.	Regular drinking water	Not reported	Early drinking: 4/100 (4%) Regular: 6/100 (6%)	Not reported	Early drinking: 1.39 ± 1.2 Regular: 7.51 ± 1.48	Not reported	Not reported
Lv et al. (2022) [16]	200 100, early drinking; 100, regular drinking water	General anaesthesia	Mallampati classification < III; mental disorders; abnormal digestive tract function; other diseases affecting observation and assessment of digestive tract function	Arthroscopic cruciate ligament reconstruction of the knee joint	Early stepped intervention with a small amount of drinking water was performed, and 37℃ normal saline was given once every 15 min, 10 mL for each of the first three times, 15 mL for each of the next three times, and 20 mL for each of the following three times.	Regular drinking water	Not reported	Not reported	Not reported	Early drinking: 6.71 ± 1.32 Regular: 8.63 ± 1.72	Early drinking: 17.53 ± 7.28 Regular: 23.67 ± 9.67	Early drinking: 50.37 ± 12.65 Regular: 59.86 ± 12.37
Gu et al. (2020) [18]	400 200, early drinking; 200, regular drinking water	General anaesthesia	Oral, nasal and pharyngeal operations; nausea and vomiting; cognitive impairment and severe complications	Non-gastrointestinal surgery	Early water intervention, the first slow injection of 5 mL, 10 min later, can be repeated once.	Regular drinking water	Not reported	Not reported	Not reported	Early drinking: 4.62 ± 1.65 Regular: 5.07 ± 2.14	Not reported	Not reported
Zhang et al. (2021) [23]	118 59, early drinking 59, regular drinking water	Regular drinking water	History of gastrointestinal disease or surgery; gastrointestinal tract or strict fasting after surgery; severe adverse anaesthetic reactions; unable to match the study	Laparoscopic surgery for gynaecological malignancies	After assessment, early drinking water was given, and a small amount of warm water was slowly dripped from the corners of the patient’s mouth using a syringe or a graduated spoon. The initial amount was 5 mL each time, and the upper limit was 100 mL per hour.	Regular drinking water	Not reported	Not reported	Not reported	Early drinking: 2.06 ± 0.64 Regular: 5.32 ± 1.51	Early drinking: 14.81 ± 3.67 Regular: 22.69 ± 6.71	Early drinking: 54.16 ± 9.11 Regular: 58.72 ± 10.35

*ASA* American Society of Anesthesiology, *PONV* postoperative nausea and vomiting

### Primary outcomes

#### Vomiting

**Fig. 4:**
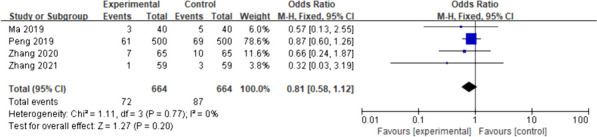
Primary outcome: vomiting. The forest plot of pooled data on changes in the incidence of vomiting in the experimental and control groups using a fixed-effects model

The four included studies [14, 15, 17, 22] showed no heterogeneity (I^2^ = 0%, *p* = 0.77); therefore, a fixed-effects model was used for meta-analysis. According to the results, there was no significant difference in the incidence of postoperative vomiting between the intervention group and the control group (OR = 0.81; 95%CI [0.58–1.12]; *p* = 0.20) (Fig. 4). The experimental and control groups had a sufficient sample size, with both including 664 patients; therefore, the possibility that early drinking water would not increase the incidence of vomiting in patients could be supported to a certain extent. However, owing to the small number of included studies assessing this outcome and the limited involvement of surgical types, further studies are needed.

#### Aspiration

**Fig. 5:**
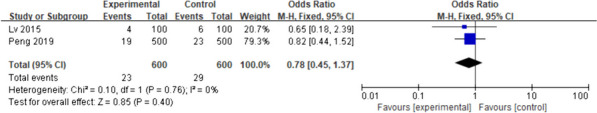
Primary outcome: aspiration. The forest plot of pooled data on changes in the incidence of aspiration in the experimental and control groups using a fixed-effects model. CI, confidence interval

Four studies [20–23] mentioned aspiration indicators. Only two studies [20, 21] were included, given that the other two studies [22, 23] did not include aspiration. I^2^ = 0% was used as the fixed-effects model for the meta-analysis. There was no significant difference in the postoperative aspiration rate between the intervention and the control groups (OR = 0.78; 95%CI [0.45–1.37]; *p* = 0.40), as shown in Fig. 5.

### Secondary outcomes

#### Nausea

**Fig. 6:**
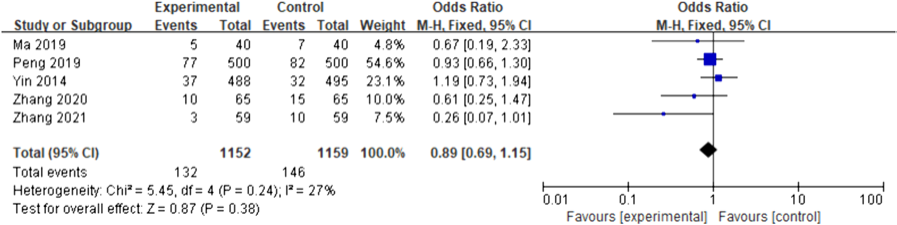
Secondary outcome: nausea. Forest plot of pooled data for nausea across studies with comparator group using a fixed-effects model. CI, confidence interval

The five included studies [14, 15, 17, 20, 22] had low inter-study heterogeneity (I^2^ = 27%, *p* = 0.24); therefore, the fixed-effects model was applied for the meta-analysis. Compared with the control group, the incidence of postoperative nausea (OR) in the intervention group was 0.89 (95%CI [0.69- 1.15]; *p* = 0.38), although the difference was not statistically significant (Fig. 6).

#### Degree of thirst

**Fig. 7:**
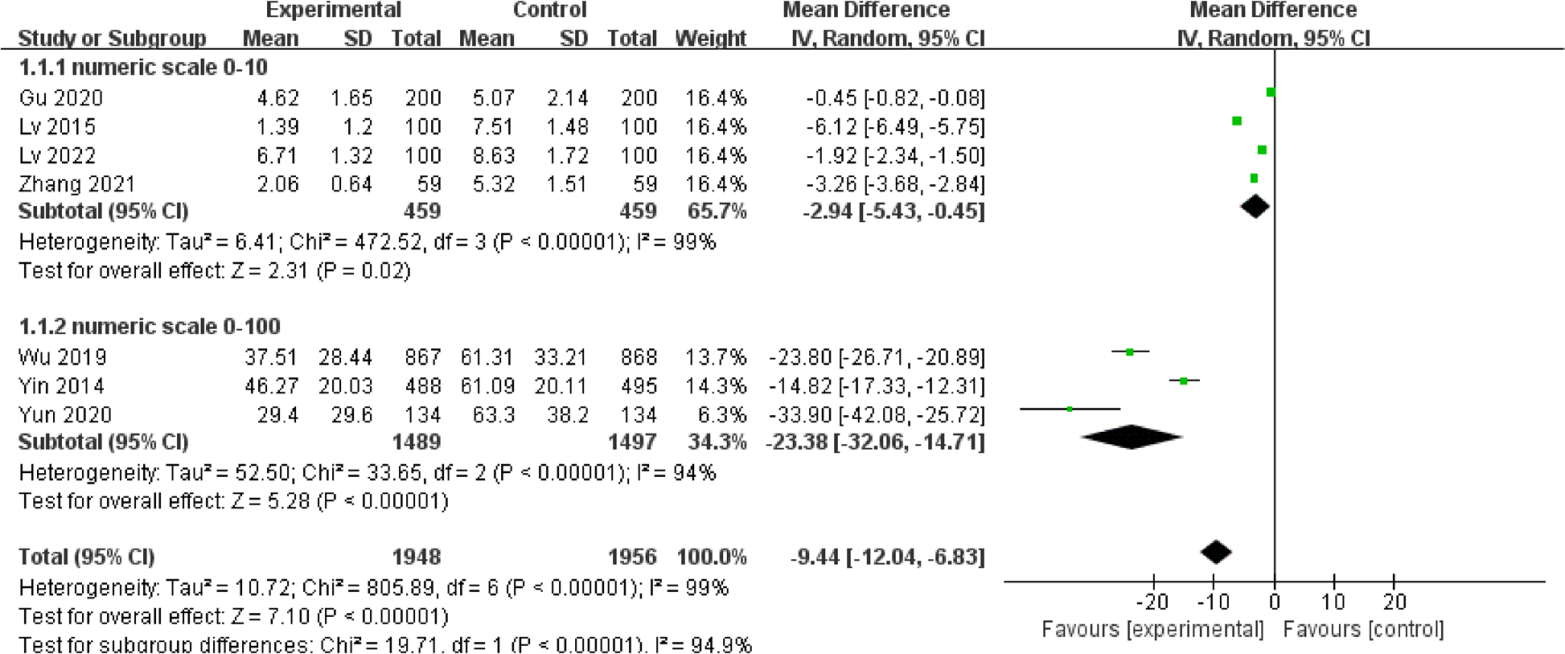
Secondary outcome: degree of thirst. Forest plot of pooled data for the degree of thirst across studies with comparator group using a random-effects model. CI, confidence interval; SD, standard deviation

Statistical heterogeneity among the seven included studies [14–18, 21, 23] was large (I^2^ = 99%; *p* < 0.01), with an MD of -9.44 (95%CI [-12.04- -6.83], *p* < 0.01) for thirst scores in the intervention group when compared with those of the control group. Subgroup analysis was performed according to the different numerical scoring criteria. The results of the subgroup analysis with a score from 0 to 10 showed that early drinking significantly improved thirst (MD = -2.94; 95%CI [-5.43- -0.45]; *p* = 0.02; I^2^ = 99%), while the results of the other subgroup with a score from 0 to 100 also revealed that early drinking significantly improved thirst (MD = -23.38; 95%CI [-32.06- -14.71]; *p* < 0.01; I^2^ = 94%), as shown in Fig. 7. Sensitivity analyses were performed for each subgroup, excluding the studies individually. No sources of heterogeneity were detected; this could be attributed to the individualisation of numbers expressing the degree of thirst for each patient according to their own comprehension, resulting in a wide variation in scores.

#### Anal exhaust time

**Fig. 8:**
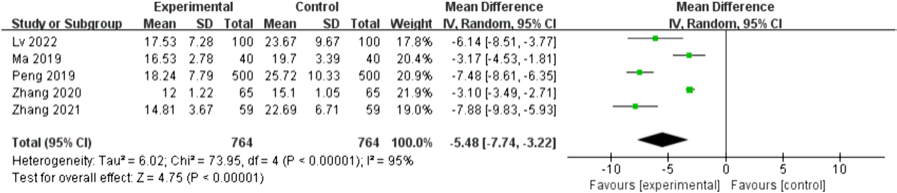
Secondary outcome: anal exhaust time. Forest plot of pooled data on anal exhaust time across studies with comparator group using a random-effects model

Five studies [16, 19, 20, 22, 23] reported the effect of early water intake on anal exhaust time in postoperative patients. The heterogeneity between the studies was high (I^2^ = 95%, *p* < 0.01); therefore, a random-effects model was used. Patients in the intervention group had a shorter anal exhaust time than patients in the control group, with a statistically significant difference (MD = -5.48; 95%CI [-7.74- -3.22]; *p* < 0.01), as shown in Fig. 8. Sensitivity analysis was performed, with the effect estimates held constant, indicating the robustness of the pooled results.

#### First defecation time

**Fig. 9:**
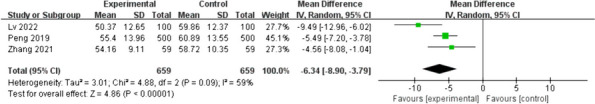
Secondary outcome: first defecation time. Forest plot of pooled data on first defecation time across studies with comparator group using a random-effects model

Three studies [16, 20, 23] included postoperative patient defaecation time as an outcome indicator; there was high heterogeneity between these studies (I^2^ = 59%; *p* = 0.09); therefore, a random-effects model was employed. Patients in the intervention group had significantly shorter postoperative defaecation times than those in the control group (MD = -6.34, 95%CI [-8.90- -3.79], *p* < 0.01), as shown in Fig. 9. A sensitivity analysis was conducted, where the effect estimates were kept constant, revealing the reliability and stability of the combined outcomes.

#### Trial sequential analysis(TSA)

The TSA results of both primary outcomes indicated that the curve was not crossed the traditional and TSA thresholds, and the cumulative sample size was not reached the expected value, requiring further research, as shown in Supplementary Figs. 1 and 2.

#### GRADE assessment

We assessed the quality of the outcome using the GRADE evaluation. The quality of the studies that were included was generally low, which led to a downgrading of the risk of bias to “serious”. Moreover, the I^2^ values for both the degree of thirst, anal exhaust time and first defecation time displayed a significant level of inconsistency, resulting in a downgrade of the inconsistency rating to “serious”. In the GRADE evaluation, except for the quality assessment of degree of thirst, anal exhaust time and first defecation time, the quality of other outcomes was moderate. Supplementary Table 1 summarized the overall results of the GRADE assessment.

## Discussion

In the present systematic review and meta-analysis, we pooled data from 10 randomised controlled studies. We found that contrary to conventional beliefs, early water intake did not increase the incidence of nausea and vomiting or the risk of aspiration in patients. Moreover, implementing early water intake in fully conscious patients post-general anaesthesia could significantly improve thirst and shorten the time to defaecation and bowel movement in accordance with ERAS.

According to conventional wisdom, early postoperative water intake can lead to nausea and vomiting, and even aspiration. Postoperative nausea and vomiting (PONV) is influenced by factors such as inhaled anaesthesia and opioid analgesics, as well as the type of surgery [24], and is not caused by fluid consumption. The main mechanism of aspiration is the relaxation of the cardiac sphincter due to general anaesthesia and suppression of the gag reflex, resulting in the regurgitation of gastric contents, causing aspiration [25]. Considering non-gastrointestinal surgery, gastrointestinal activity in patients who received general anaesthesia can return to baseline levels at an early stage, and multiple small amounts of gradual fluid consumption can allow physiological adaption of gradual gastrointestinal function recovery post-general anaesthesia without increasing the incidence of postoperative complications [26]. The Guidelines for Perioperative Fasting and Water Fasting in Adults and Children developed by the European Society of Anesthesia also recommends drinking water as early as possible, according to the patient’s subjective desire, to promote gastrointestinal motility and reduce the incidence of nausea and vomiting [27]. Considering the literature included in the current meta-analysis, four [14, 15, 17, 22] studies found no statistically significant differences in the incidence of nausea and vomiting during post anaesthesia care unit (PACU) stay and after return to the ward with early water intake between the intervention and control groups. Two studies [20, 21] noted that early water intake did not increase the risk of patient aspiration. It should be noted that the present study involved limited types of surgery, and the timing of early water intake and incidence of adverse postoperative complications need to be further explored for other types of surgery, particularly gastrointestinal surgery.

Thirst is defined as the conscious desire to drink water, which is a compensatory mechanism that allows an organism to restore its water balance [28]. Preoperative fasting and abstinence from food and fluid, anaesthetic medications, tracheal intubation, and intraoperative bleeding can exacerbate thirst [29, 30]. Thirst in perioperative patients is a sign and symptom of imbalance and intense discomfort, causing discomfort in patients during recovery from anaesthesia, leading to a series of organismal stress reactions such as negative emotions and increased risk of wound bleeding, markedly prolonging the patient’s postoperative recovery time and failing to achieve comfortable care [30–32], thereby necessitating the attention and assistance of medical personnel. Lee and others [33] have shown that moderate to severe postoperative thirst was common in PACU, a finding that is consistent with a cross-sectional observational study conducted at the National Health Service Hospital in the United Kingdom [34]. The most direct and effective way to alleviate thirst after regaining consciousness from general anaesthesia is to drink water early during the postoperative period [35]. In one included study, patients who administered water early had significantly lower thirst scores and a correspondingly lower incidence of thirst than patients who routinely abstained from water. Accordingly, early water intake largely reduces postoperative discomfort in patients and improves their satisfaction with medical visits.

Postoperative anal exhaust time and time to defaecation are clinically important indicators of gastrointestinal function recovery; therefore, these two indicators were used as outcome indicators in the present study. Herein, we found that early oral administration could promote the recovery of gastrointestinal function in postoperative patients, which is in line with the ERAS concept and consistent with the results of related studies [36, 37]. Water is considered the mildest mechanical stimulus and adequate hydration is crucial for bodily functions [38]. Early water consumption can stimulate the oral cavity and gastrointestinal tract, promote the secretion of digestive juices through the neurohumoral reflex, increase gastrointestinal tract peristalsis, and promote the recovery of gastrointestinal function [39, 40].

Early assessment is the most important prerequisite for implementing drinking water to ensure patient safety. The guidelines for post-anaesthesia care proposed by the American Society of Anesthesiologists state that the safety criteria used to assess patients after anaesthesia include the level of consciousness, airway patency, respiratory rate, and blood oxygen saturation to avoid potential complications [41]. The literature included in the current study states the assessment measures that were implemented to guarantee patient safety. In two studies [14, 15], patients in the intervention group were assessed as fully awake, with stable vital signs, muscle strength grade 5, and good recovery of cough and gag reflexes when water was administered. Two studies [17, 20] employed the Steward or Aldrete rating scales to assess whether patients were fully awake and eligible for early water intake. Larger multicentre studies are needed to develop a uniform assessment tool for early water intake during the postoperative awakening period after general anaesthesia that would meet the characteristics of all populations.

Our study had some limitations. The present study did not include additional outcome indicators such as patient comfort, willingness to drink, and abdominal distension. In addition, there were inconsistencies in recording outcome indicators in some studies, with some reports using measures while others using counts; this made it impossible to include the combined literature owing to difficulties in data extraction. Therefore, the number of studies included in the present analysis for certain analysed indicators was insufficient to determine whether publication bias had occurred. The literature not included in the current analysis, owing to data extraction issues, could also have impacted study results, leading to bias. The literature included in the current analysis was mainly focused on adult patients, with no statistical analysis conducted on special populations such as children and patients with acute and critical illnesses. The type of surgery involved was not comprehensive, and additional factors affecting adverse patient outcomes were not explored. Different hospitals in different countries have distinct strategies for implementing early water intake, which could impact the accuracy of the present meta-analysis. Therefore, more randomised controlled studies assessing special populations and different diseases are needed to comprehensively clarify the safety and feasibility of early water intake.

## Conclusion

The results of the present meta-analysis revealed that early water intake during the postoperative awakening period in patients who had received general anaesthesia could relieve thirst without increasing the incidence of adverse postoperative complications such as nausea, vomiting, and aspiration, and this could be applied in clinical practice. Owing to the heterogeneity in the included literature, a high-quality, large-sample, multicentre, randomised controlled study is needed. Moreover, the implementation time and amount of early water intake vary across hospitals, and more rigorously designed randomised controlled trials are needed to clarify the timing and amount of early water intake.

### Supplementary Information

**Supplementary Material 1.**

## Data Availability

The datasets used and/or analysed during the current study are available from the corresponding author on reasonable request.

## Abbreviations

ERAS: Enhanced recovery after surgery
PONV: Postoperative nausea and vomiting
PACU: Post anaesthesia care unit
CNKI: China National Knowledge Infrastructure
ASA: American Society of Anesthesiology
CI: Confidence interval
MD: Mean difference
OR: Odds ratio
I^2^: I-squared
TSA: Trial sequential analysis
