# Supplementary material for: Timing of early water intake post-general anaesthesia: a systematic review and meta-analysis
Source: BMC Anesthesiol. 2024 Apr 9;24:135. doi: 10.1186/s12871-024-02520-x (PMC11003094; doi:10.1186/s12871-024-02520-x)
Supplement: Supplementary file 1 — Supplementary Material 1. [file 12871_2024_2520_MOESM1_ESM.pdf]

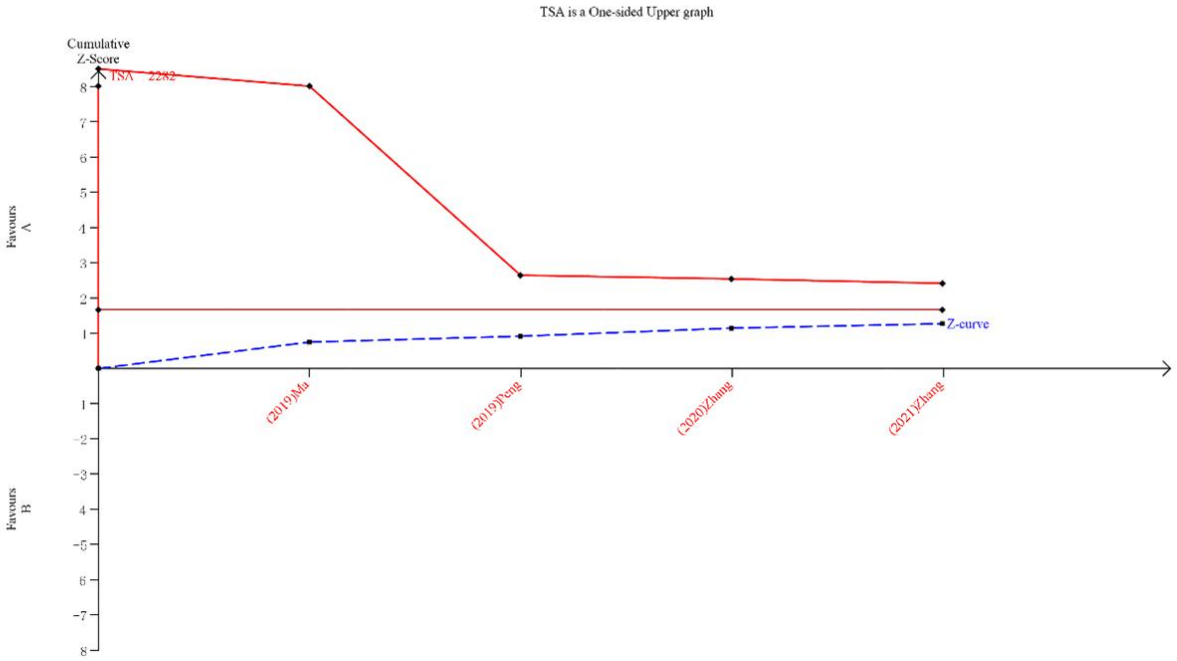

Supplementary Fig.1. Trial sequential analysis for vomiting.

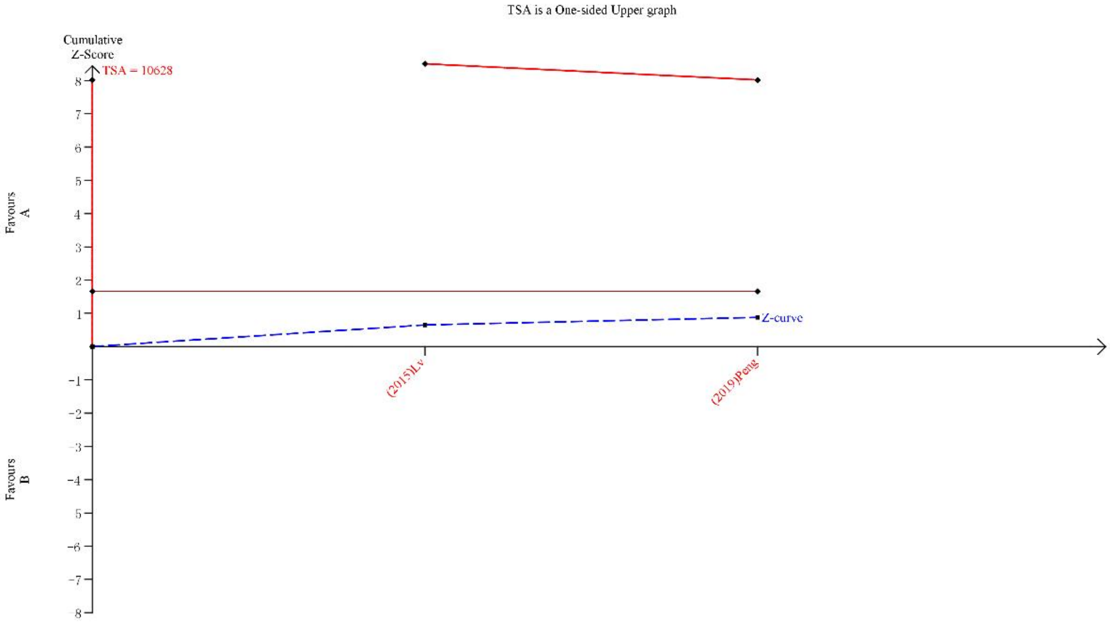

Supplementary Fig.2. Trial sequential analysis for aspiration.

Supplementary Table 1. Results of GRADE evaluation.

Outcome	MD/OR (95%CI)	Number of studies	Quality of evidence	Reasons

Vomiting	0.81(0.58-1.12)	4	⊕⊕⊕◯ moderate	Risk of bias was “serious”.
Aspiration	0.78(0.45-1.37)	2	⊕⊕⊕◯ moderate	Risk of bias was “serious”.
Nausea	0.89(0.69-1.15)	5	⊕⊕⊕◯ moderate	Risk of bias was “serious”.
Degree of thirst	-9.44 (-12.04- -6.83)	7	⊕⊕◯◯ low	Risk of bias was “serious”; inconsistency was “serious”.
Anal exhaust time	-5.48(-7.74- -3.22)	5	⊕⊕◯◯ low	Risk of bias was “serious”; inconsistency was “serious”.
First defecation time	-6.34(-8.90- -3.79)	3	⊕⊕◯◯ low	Risk of bias was “serious”; inconsistency was “serious”.

MD, mean difference; OR, odds ratio;CI, confidence interval
